# Noisy galvanic vestibular stimulation has a greater ameliorating effect on posture in unstable subjects: a feasibility study

**DOI:** 10.1038/s41598-019-53834-7

**Published:** 2019-11-20

**Authors:** Chisato Fujimoto, Makoto Kinoshita, Teru Kamogashira, Naoya Egami, Takuya Kawahara, Yukari Uemura, Yoshiharu Yamamoto, Tatsuya Yamasoba, Shinichi Iwasaki

**Affiliations:** 1 Department of Otolaryngology and Head and Neck Surgery, Graduate School of Medicine, The University of Tokyo 7-3-1, Hongo, Bunkyo-ku, Tokyo 113-8655 Japan; 2 Department of Otolaryngology, Tokyo Teishin Hospital, 2-14-23, Fujimi, Chiyoda-ku, Tokyo 102-8798 Japan; 3 Biostatistics Division, Clinical Research Support Center, The University of Tokyo Hospital 7-3-1, Hongo, Bunkyo-ku, Tokyo 113-8655 Japan; 4 Educational Physiology Laboratory, Graduate School of Education, The University of Tokyo 7-3-1, Hongo, Bunkyo-ku, Tokyo 113-0033 Japan

**Keywords:** Peripheral nervous system, Neurology

## Abstract

Ameliorating effect of noisy galvanic vestibular stimulation (nGVS) on posture varies among subjects. In this feasibility study, we investigated the association between original postural instability and the ameliorating effect of nGVS on posture. Data were collected in a previously published study. Thirty healthy elderly were recruited. Two nGVS sessions (30 min or 3 h) were performed in a randomised order. The optimal intensity of nGVS, the most effective intensity for improving posture, was determined before each session. Posture was measured for 30 s during and after nGVS in the eyes-closed/foam rubber condition. The velocity, envelopment area, and root mean square of the centre of pressure movement without nGVS were significantly larger in the group with an optimal intensity than those in the group without an optimal intensity. There was a significant positive correlation between these values and the long-term ameliorating effects. The ratio of the values in the eyes-closed/foam rubber condition to those in the eyes-open condition was significantly larger in the group with an optimal intensity, and had a significant correlation with the long-term ameliorating effects. The ameliorating effects are greater in subjects who were originally unstable and in those whose postural stability was relatively independent of vestibular input.

## Introduction

Noisy galvanic vestibular stimulation (nGVS) is a procedure that applies electrical current as zero-mean current noise to the vestibular end organs and their afferent nerves through electrodes placed bilaterally over the mastoid process. An imperceptible level of nGVS facilitates the processing of subthreshold stimuli in neural systems, such as the autonomic, motor or postural control systems^1–4^. With regard to postural control, stability while standing was improved during the application of an optimal level of nGVS in healthy subjects as well as in patients with bilateral vestibulopathy (BV)^2,5–8^. Walking stability was also improved during nGVS application in healthy subjects and in BV patients^2,9–11^. The proposed mechanism behind these effects is stochastic resonance (SR), in which the existence of an optimal level of noise can enhance subthreshold signals in a non-linear system^12,13^.

We recently reported that nGVS has a post-stimulation effect on postural improvement in healthy elderly subjects and in BV patients^1,14^. A 30-min application of nGVS led to a sustained postural improvement that lasted for several hours, even after the cessation of the stimulus^1,14^. Patients with peripheral vestibulopathy have been reported to show an increase in the mid-high frequency component of COP movement while standing on a stable platform^15–17^. In our previous study, a shift to the lower frequency component was observed in BV patients during the post-stimulation period (PST) of nGVS^14^. This newly discovered post-stimulation effect of nGVS may have an association with neuroplasticity in the vestibular system. Aging reduces the function of all components of the postural control system^18^. This decline in postural control with age can be a significant cause of falls^19^. These nGVS effects can contribute to the improvement of postural stability not only for patients with peripheral vestibular disorders but also for the elderly.

However, the effect of nGVS on the improvement of body balance in healthy subjects and patients with vestibulopathy is not uniform among subjects^1,2,5,8^. We previously examined postural stability in healthy subjects during 30-s nGVS by measuring 3 representative parameters: the mean velocity, the envelopment area, and the root mean square (RMS) of the movement of the centre of pressure (COP), and showed that most subjects showed postural improvement as a result of nGVS in all 3 parameters^1,2^. However, the degree of improvement in postural stability was different among subjects, and in a certain portion of subjects, nGVS had little effect on postural stability.

We hypothesised that the amount of postural instability of the subject before application of nGVS (original postural instability) affects the ameliorating effect of nGVS. We have previously designed a feasibility study to examine the long-term effects of nGVS on postural stability in healthy elderly before conducting a pivotal study to examine the effect of nGVS on postural stability in patients with vestibulopathy^1^. Here, we use data from the study and newly investigate the association between the original postural instability and the ameliorating effect of nGVS on postural stability in healthy elderly adults.

## Results

### Optimal intensity of nGVS

The 3 COP parameters, velocity, area and RMS were measured using posturography. We performed two experimental sessions (Session 1 and Session 2) to investigate the long-term effect of nGVS^2,4^. Before starting each session, the value of each COP parameter in the eyes-open without nGVS condition was measured for 30 s, and then the optimal intensity of nGVS was determined. To determine the optimal intensity, first the value of each COP parameter in the eyes-closed/foam rubber condition without nGVS was measured and this was defined as the baseline value. Then, the value of each COP parameter in the eyes-closed/foam rubber condition with the 30-s nGVS application was measured. The optimal intensity was defined as the intensity at which the value measured during the 30-s nGVS application was smaller than the baseline value simultaneously in all of the 3 COP parameters^1^. In Session 1, optimal intensity of nGVS was applied to subjects for two 30 min stimulation periods (STs) with a 4-h interval. In Session 2, optimal intensity of nGVS was applied to subjects for 3 h and the subjects were monitored after the cessation of the stimulus for 4 h.

Thirty participants were randomly assigned to first undergo Session 1 and then to undergo Session 2 (Group A), or to first undergo Session 2 and then to undergo Session 1 (Group B). We measured postural stability for 30 s in each subject twice on separate days before and during the application of graded intensities of nGVS to determine the optimal intensity of nGVS (Supplementary Table S1). Out of the 30 subjects, twenty had an optimal intensity in both of the two measurement periods. Seven had an optimal intensity in only one of the two periods. One subject did not have an optimal intensity in either period. Two subjects had an optimal intensity in one measurement period but due to failure of the device an optimal intensity could not be obtained during the second measurement period (before Session 2). An optimal intensity was obtained in 49 of the 58 measurement periods (84%) (Supplementary Table S1). The mean of the optimal intensity was 178.8 (±9.1) μA.

**Figure 1:**
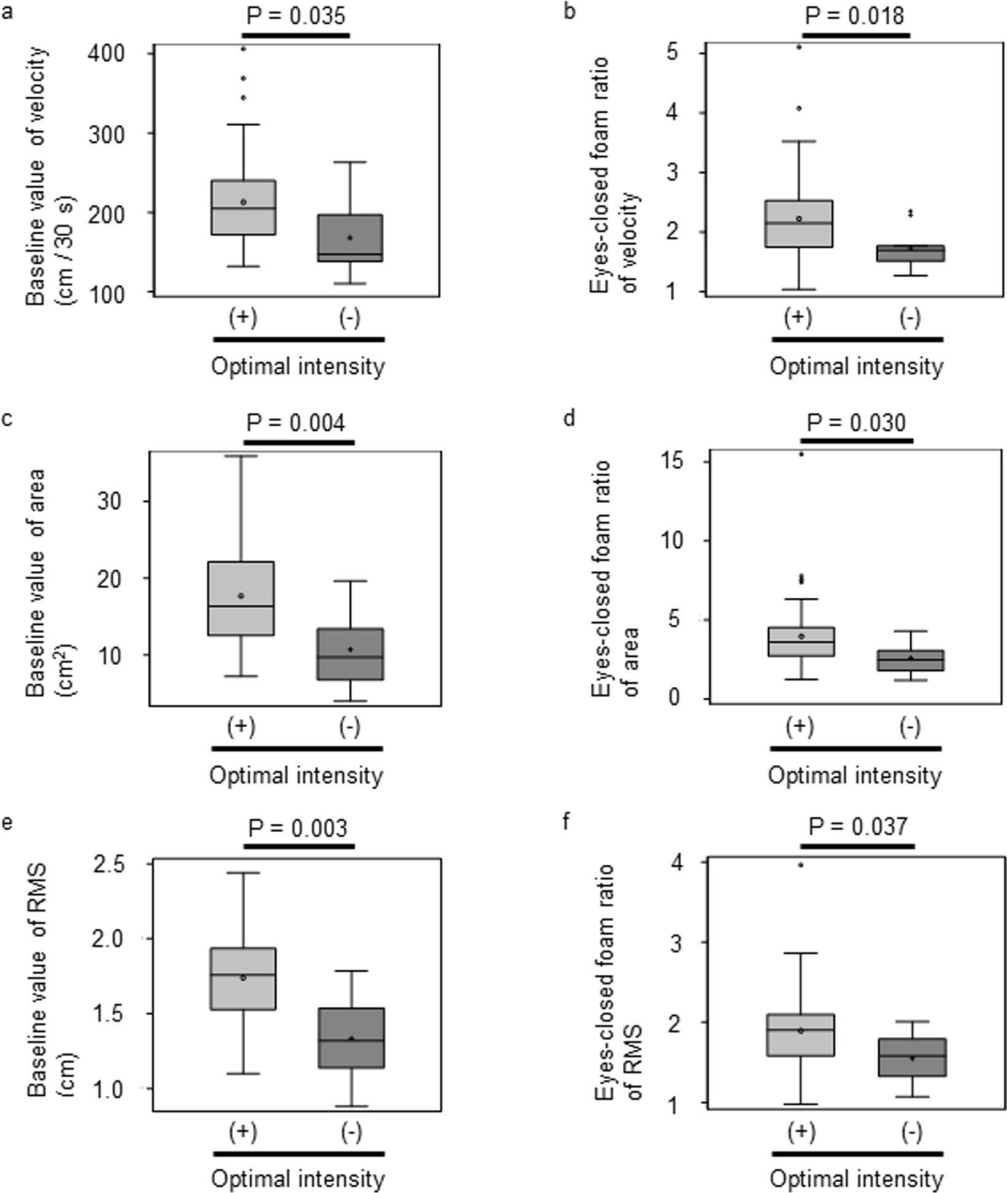
Box-and-whisker plots of original postural instability in a group with optimal intensities (n = 49) and a group without optimal intensities (n = 9). **(a)** Baseline value of the velocity. **(b)** Eyes-closed foam ratio of the velocity. **(c)** Baseline value of the area. **(d)** Eyes-closed foam ratio of the area. **(e)** Baseline value of the RMS. **(f)** Eyes-closed foam ratio of the RMS by the measurement times with or without optimal intensity.

First, we analyzed the association between original postural instability and the presence of an optimal intensity of nGVS. As we found that there is evidence for non-normality of eyes-closed foam ratio of area or RMS, we conducted non-parametric statistical methods. The baseline values of postural stability (velocity, area, and RMS of COP) in the group with an optimal intensity were significantly larger than those in the group without an optimal intensity [P = 0.035 (velocity, Fig. 1a), P = 0.004 (area, Fig. 1c), P = 0.003 (RMS, Fig. 1e), Mann-Whitney U test]. We also analyzed the eyes-closed foam ratio of the COP parameters, which is the ratio of the values in the eyes-closed/foam rubber condition to those in the condition with the eyes-open standing on a firm platform. The smaller this ratio is, the larger the vestibular dependence of postural stability is^20^. The eyes-closed foam ratio of the COP parameters in the group with an optimal intensity was also significantly larger than that in the group without an optimal intensity [P = 0.018 (velocity, Fig. 1b), P = 0.030 (area, Fig. 1d), P = 0.037 (RMS, Fig. 1f), Mann-Whitney U test].

### Ameliorating effect during long-term nGVS

**Figure 2:**
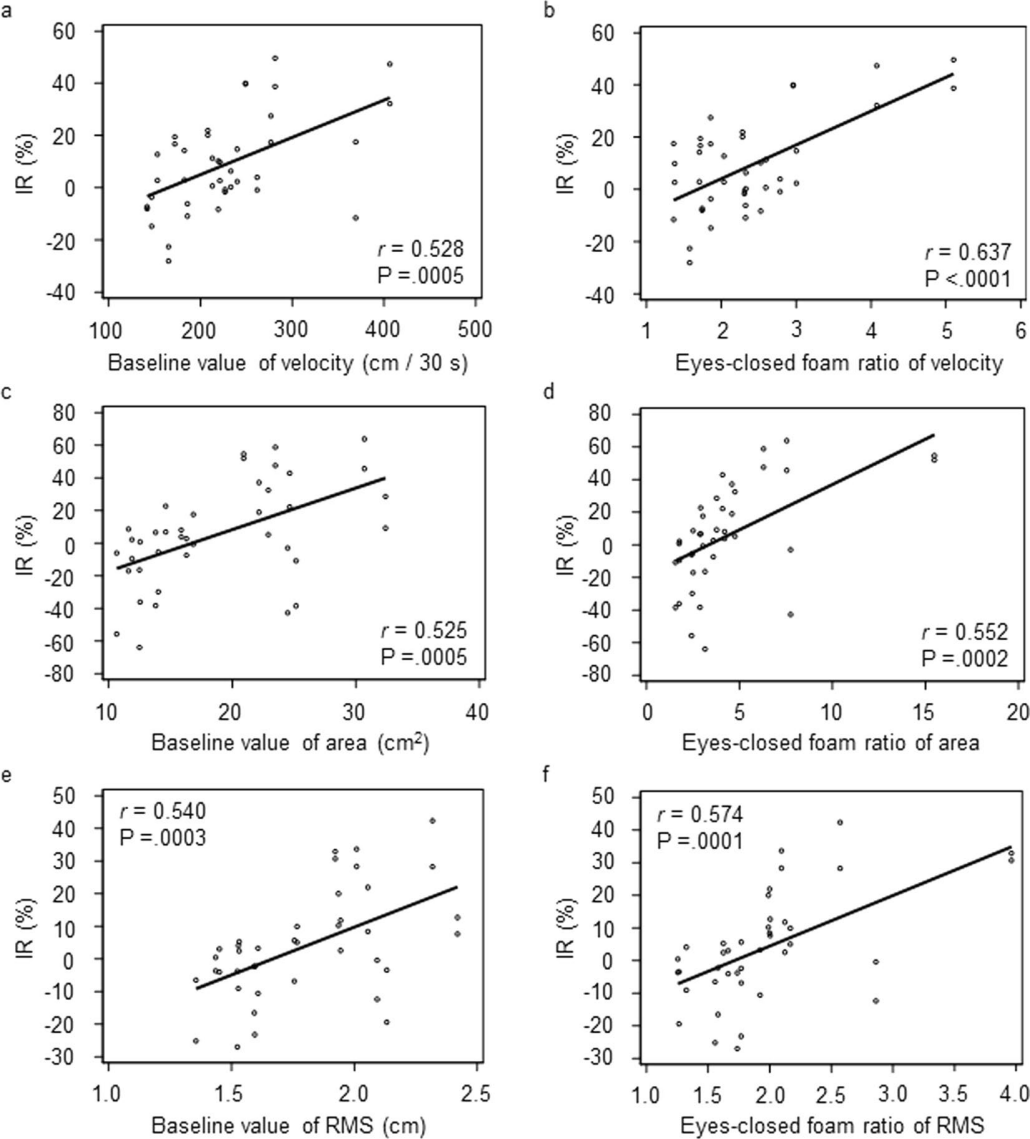
Relationship between original postural instability and the effect of nGVS on postural stability during the 3-h stimulation (n = 40). **(a)** Baseline value of the velocity. **(b)** Eyes-closed foam ratio of the velocity. **(c)** Baseline value of the area. **(d)** Eyes-closed foam ratio of the area. **(e)** Baseline value of the RMS. **(f)** Eyes-closed foam ratio of the RMS. IR, improvement rate; r denotes Pearson’s correlation coefficient and associated p-values are shown.

Next, we analyzed the association between original postural stability and the effect of nGVS during the 3-h stimulation in 20 subjects who had an optimal intensity before both sessions. We defined the improvement rate (IR) (%) as 100 (1 – normalised ratio), where the normalised ratio is the ratio of the value at the measurement period to that at baseline. There were moderate, but significant correlations between the baseline values of postural stability and their improvement during long-term nGVS [r = 0.528 (velocity, Fig. 2a), r = 0.525 (area, Fig. 2c), r = 0.540 (RMS, Fig. 2e), Pearson’s correlation coefficient]. Similarly, there were moderate correlations between the eyes-closed foam ratio of the COP parameters and their improvement during long-term nGVS [r = 0.637 (velocity, Fig. 2b), r = 0.552 (area, Fig. 2d), r = 0.574 (RMS, Fig. 2f), Pearson’s correlation coefficient]. Mixed-effects model analyses demonstrated that the larger the baseline values of postural stability were, the greater the improvement of postural stability was as a result of long-term nGVS [P = 0.011 (velocity), P = 0.007 (area), P = 0.007 (RMS), Table 1]. The analyses also showed that the larger the eyes-closed foam ratio of the postural parameters were, the greater the postural improvement was [P = 0.001 (velocity), P = 0.004 (area), P = 0.003 (RMS), Table 1]. We conducted the mixed-effects model analysis by adding heights and weights in the fixed factors, and found that their p-values were larger than 0.1. For example, F-values for height and weight were 0.06 (P = 0.815) and 0.21 (P = 0.650), respectively. Therefore, we considered that the subject’s height and weight scarcely affect the effect of nGVS, or these factors indirectly affect the effect of nGVS through baseline posturographic data.

**Table 1:** Mixed-effects model analyses to explore the association between the original postural instability and the effect of nGVS on postural stability during the 3-h stimulation.

Parameter	Fixed effect	Estimate	Standard error	F value	P value
Velocity	Baseline value (by 10 cm / 30 s)	1.43	0.50	8.00	0.011
	Time			1.96	0.178
	Eyes-closed foam ratio	12.93	3.39	14.58	0.001
	Time			1.96	0.178
Area	Baseline value	2.55	0.83	9.33	0.007
	Time			0.01	0.923
	Eyes-closed foam ratio	5.55	1.68	10.90	0.004
	Time			0.01	0.923
RMS	Baseline value	29.27	9.55	9.39	0.007
	Time			0.25	0.623
	Eyes-closed foam ratio	15.50	4.59	11.38	0.003
	Time			0.25	0.623

### Post-stimulation ameliorating effect of nGVS

**Figure 3:**
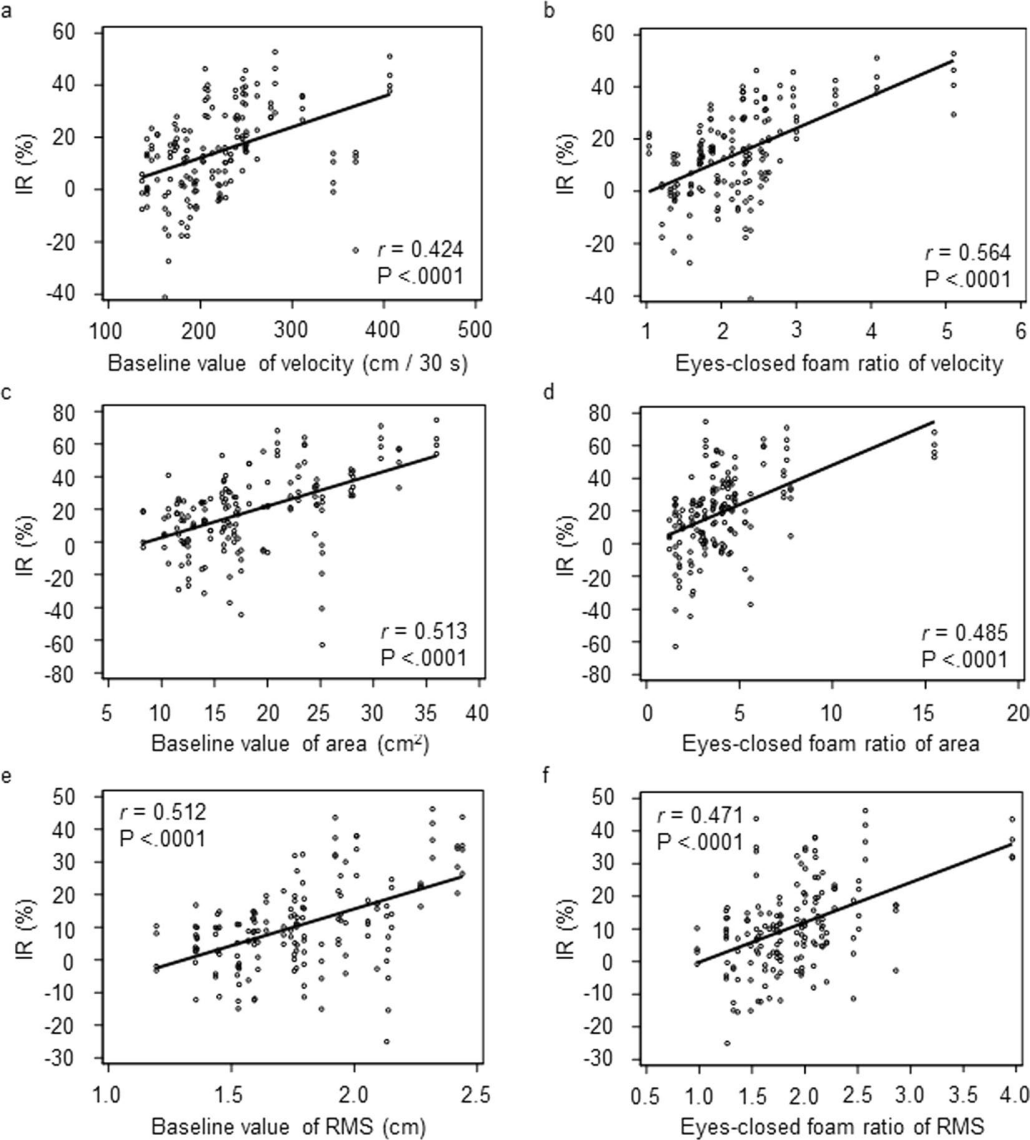
Relationship between original postural instability and the long-term post-stimulation effect of nGVS on postural stability (n = 160). **(a)** Baseline value of the velocity. **(b)** Eyes-closed foam ratio of the velocity. **(c)** Baseline value of the area. **(d)** Eyes-closed foam ratio of the area. **(e)** Baseline value of the RMS. **(f)** Eyes-closed foam ratio of the RMS. IR, improvement rate; r denotes Pearson’s correlation coefficient and associated p-values are shown.

Finally, we analyzed the association between original postural stability and the long-term post-stimulation ameliorating effect of nGVS on postural stability. Posturographic data at PST 30 min, 1 h, 2 h and 3 h in the first 30-min nGVS and in the 3-h nGVS were used. Data in the second 30-min nGVS were not used due to the influence of the cumulative effect. There were moderate, but significant correlations between the baseline values of postural stability and the post-stimulation ameliorating effect of nGVS [r = 0.424 (velocity, Fig. 3a), r = 0.513 (area, Fig. 3c), r = 0.512 (RMS, Fig. 3e), Pearson’s correlation coefficient], and between the eyes-closed foam ratio of the postural parameters and the post-stimulation ameliorating effect [r = 0.564 (velocity, Fig. 3b), r = 0.485 (area, Fig. 3d), r = 0.471 (RMS, Fig. 3f), Pearson’s correlation coefficient]. Mixed-effects model analyses demonstrated that the larger the baseline value was, the greater the postural improvement was [P < 0.0001 (velocity, area, RMS), Table 2]. The analyses also showed that the larger the eyes-closed foam ratio, the greater the postural improvement was [P < 0.0001 (velocity, area, RMS), Table 2]. We conducted the mixed-effects model analysis by adding heights and weights in the fixed factors, and found that their p-values were larger than 0.1. For example, F-values for height and weight were 0.65 (P = 0.432) and 0.11 (P = 0.742), respectively. Therefore, we considered that the subject’s height and weight scarcely affect the effect of nGVS, or these factors indirectly affect the effect of nGVS through baseline posturographic data.

**Table 2:** Mixed-effects model analyses to explore the association between the original postural instability and the long-term post-stimulation effect of nGVS on postural stability.

Parameter	Fixed effect	Estimate	Standard error	F value	P value
Velocity	Baseline value (by 10 cm / 30 s)	2.35	0.23	105.91	<0.0001
	Time			0.95	0.422
	Session			1.09	0.309
	Time * Session			0.22	0.884
	Eyes-closed foam ratio	11.00	1.61	46.83	<0.0001
	Time			0.66	0.582
	Session			1.02	0.325
	Time * Session			0.15	0.929
Area	Baseline value	2.64	0.26	104.62	<0.0001
	Time			1.88	0.143
	Session			0.76	0.396
	Time * Session			0.28	0.839
	Eyes-closed foam ratio	5.13	0.80	41.50	<0.0001
	Time			1.29	0.286
	Session			0.12	0.728
	Time * Session			0.19	0.901
RMS	Baseline value	34.60	2.99	133.87	<0.0001
	Time			1.68	0.181
	Session			0.00	0.949
	Time * Session			0.34	0.793
	Eyes-closed foam ratio	14.41	2.12	46.03	<0.0001
	Time			1.08	0.367
	Session			0.21	0.652
	Time * Session			0.22	0.882

## Discussion

In the present study, we revealed that pre-existing instability had a significant association with the presence of an optimal intensity. The more unstable the original posture was, the greater the long-term ameliorating effect of nGVS on postural stability was, both during the stimulus and after cessation of the stimulus. An increased ratio of the eyes-closed/foam rubber condition to the eyes-open condition had a significant association with the postural improvement after nGVS.

We have shown that an optimal intensity of nGVS causes the simultaneous improvement in velocity, area and RMS of the COP movement in most of the subjects^1,2^. In the present study, an optimal intensity was present in 84% of the measurement periods^1^, and the current value of the optimal intensity was close to previous studies^2,5,9^. On the other hand, a further increase of nGVS intensity degrades stability causing an increase in the value of these posturographic parameters^1,2^. The effect of nGVS on postural improvement varied among subjects^1,2^. The factors that regulate individual differences in the effect of nGVS have not been elucidated so far. Our present study demonstrates that original postural instability has a significant association with the presence of an optimal intensity. The mechanism underlying the ameliorating effect of nGVS on postural stability during the stimulus is thought to be SR. In SR, an optimal amount of noise added to a non-linear system enhances information processing whereas a further increase in noise intensity leads to degradation of the content processing^12,13^. In the present study, we revealed that an SR-like phenomenon in the effect of nGVS on postural stability, which is the presence of an optimal intensity, was influenced by the original postural instability of the subject. We have previously shown that nGVS at the optimal intensity for 3 h had little effect on improving postural stability during the stimulation^1^. In the present study, we found that the more unstable the original posture was, the greater the long-term effect of nGVS at the optimal intensity on postural improvement was during the stimulation. It has been shown that in the human postural control system vestibular information is dominant to somatosensory information when there is a relatively large body sway^21^. It is possible that the SR-based effects of nGVS on vestibular afferents might be masked in subjects with smaller postural sway in the eyes-closed/foam rubber condition. Another possible reason that the posture of subjects without an optimal intensity was originally stable is that it was already optimised with noise inherent in the nervous system related to postural control. One previous report showed that the ameliorating effect of 400 µA nGVS on postural stability was large in originally unstable elderly subjects^6^, supporting our hypothesis, although it is unclear whether the intensity of 400 µA was optimal for these subjects.

Moreover, this greater ameliorating effect in originally more unstable subjects was shown not only during the stimulus but also after the cessation of the stimulus. Elderly subjects with symptoms of unsteadiness are likely to have more severe postural instability than the healthy elderly subjects in the present study, and the ameliorating effect of nGVS can be expected to be more in these subjects. Our results will be helpful as preliminary data for applying nGVS to the treatment of balance disorders in the elderly and patients with vestibulopathy.

The present study showed that an increase of the eyes-closed foam ratio had a significant association with the improvement due to nGVS. The postural control system coordinates vestibular, somatosensory and visual inputs with outputs to the musculoskeletal system at the central nervous system in order to maintain balance^22^. Several clinical tests such as dynamic posturography using a movable platform and posturography using a foam rubber surface (foam posturography) have been developed to perturb standing posture by manipulating the relative contributions of visual, somatosensory, and vestibular inputs^20,23–26^. Under the eyes-closed/foam rubber condition in a standing posture, subjects have to maintain their balance depending primarily on vestibular input due to reductions in visual and somatosensory inputs. The subjects with higher eyes-closed foam ratios are considered to be strongly influenced by reductions in visual and somatosensory inputs, suggesting that these subjects have a relatively low dependence on vestibular input in postural control^20^. Our results suggest that nGVS is effective in subjects with a low dependence on vestibular information which is consistent with the results of our previous studies showing that nGVS is effective in patients with vestibular dysfunction^2,14^.

The present study has several limitations. First, this was not a placebo-controlled study to examine the long-term effects of nGVS on postural stability. This study was designed as a feasibility study to examine the long-term effects in healthy elderly before conducting a pivotal study, a double-blind placebo-controlled crossover study to examine the effect of nGVS on postural stability in patients with vestibulopathy. Habituation or fatigue effects due to the repeated measurements of postural stability may influence the results of the long-term effect of nGVS on postural stability. However, the data that we previously reported showed that the ameliorating effect decreased after PST 2 h in both Session 1 and Session 2^1^. Additionally, our recent study showed that the improvement in the normalized ratios of velocity decreased after PST 4 h in the 30-min nGVS sessions in BV patients^14^. These results cannot be explained by the habituation effect. This suggests that an attenuated post-stimulation effect is present. Furthermore, in the present study, the second 30-min nGVS application after 4-h interval in Session 1 also caused an improvement in postural stability, and this effect is unlikely to the fatigue effect. Second, we only analyzed the ameliorating effect of nGVS on static posture in the present study. Further studies are needed to evaluate individual differences in the effect of nGVS on dynamic gait performance. Third, seven subjects had an optimal intensity in one measurement period but not in the other. We demonstrated that the baseline value and the eyes-closed foam ratio were significantly larger in the group with an optimal intensity of nGVS. However, it is still unclear why there are intraindividual differences in the presence of an optimal intensity. Fourth, in the present study, we did not evaluate cognitive function in the elderly subject. Postural control of the elderly is known to be affected by cognitive function, and a novel approach to the treatment of balance disorder has recently been considered for the elderly with cognitive decline that may hinder the effect of rehabilitation^27,28^. The cognitive function of the subject may affect the ameliorating effect of nGVS. Fifth, we did not perform vestibular function tests in the subjects included in the present study. Since we recruited subjects who had not had episodes of vertigo/dizziness or hearing loss, we assumed normal vestibular function in these subjects. However, it is possible that some of these subjects might have latent vestibular dysfunction.

In conclusion, the ameliorating effect of nGVS on postural stability is greater in subjects who were originally more unstable, both during the stimulus and after the cessation of the stimulus. Subjects with a lower dependence on vestibular inputs show greater ameliorating effects of nGVS.

## Methods

### Data used in the present study

We used data from previously reported clinical research^1^ and newly investigated the association between postural instability before application of nGVS and the ameliorating effect of nGVS on postural stability in the healthy elderly subjects. The purpose of this study is totally different from that of our previous study.

### Research ethics

This study was conducted in accordance with the Declaration of Helsinki. All procedures in this trial were approved by the Institutional Review Board of the University of Tokyo Hospital (P2114052-11Y). This trial was registered with the University Hospital Medical Information Network (UMIN) Clinical Trials Registry (UMIN-CTR: UMIN000016054; date defining the periods of recruitment and follow-up: December 24, 2014; date of registration: December 25, 2014). Written informed consent was obtained by all subjects.

### Subjects

Thirty healthy subjects (17 males and 13 females; mean age 67.0 [±1.7] years) were recruited in this trial^1^. Exclusion criteria were as follows: episodes of vertigo/dizziness, hearing loss other than age-related hearing loss, ear diseases, orthopedic diseases, cardiovascular diseases, psychiatric diseases, malignant tumor, use of tranquillizers or antidepressants, alcohol consumption after 22:00 on the day before testing, presence of metal objects in the body, and difficulty in walking without assistance.

### Posturography

Posturography was performed on a foam rubber surface using a Gravicorder GP-5000 (Anima Co. Ltd., Tokyo, Japan) containing vertical force transducers at a sampling frequency of 20 Hz. The foam rubber surface was made of natural rubber (tensile strength, 2.1 kgf/cm2; stretch percentage, 110%; thickness, 3.5 cm). Two-legged stance tasks were performed by the subjects in three conditions: eyes-open without nGVS, eyes-closed on foam rubber without nGVS, eyes-closed on foam rubber with nGVS. The mean velocity of the COP movement (velocity), the envelopment area traced by the COP movement (area), and the RMS of the COP distance moved were measured.

### nGVS

nGVS was applied with electrodes placed bilaterally over the mastoid process by a portable stimulator (112 × 67 × 28 mm; 200 g including dry cells)^2,4^. Waveforms were digitally stored and converted from digital-to-analog at 20 Hz. Zero-mean white noise GVS that ranged from 0.02 to 10 Hz was used in the present study. The white noise waveform had a duration of 204.8 sec and was continuously repeated during the tests.

### Procedures

Throughout the present study, when we performed a 30-s postographic measurement, the subject was instructed to perform a two-leg stance task for 45 s, and we measured postural stability of the subject from the 15-s point for 30 s. We performed two experimental sessions (Session 1 and Session 2) separated by a 7-day interval to investigate the long-term effect of nGVS. Before starting each session, the value of each COP parameter in the eyes-open without nGVS condition was measured for 30 s, and then the optimal intensity of nGVS was determined. To determine the optimal intensity, first the value of each COP parameter in the eyes-closed/foam rubber condition without nGVS was measured and this was defined as the baseline value. Then, the value of each COP parameter in the eyes-closed/foam rubber condition with the application of nGVS for 30 s was measured at the peak amplitudes of 50, 100, 200, 300, and 500 μA. The interval of nGVS application was set at 2 min. The optimal intensity of nGVS was defined as the intensity at which the value measured during the 30-s nGVS application was smaller than the baseline value simultaneously in all of the 3 posturographic parameters^1^. If a subject felt any sensation during nGVS application at a certain intensity, this intensity was rejected as the optimal intensity. In Session 1, optimal intensity nGVS was applied to subjects for two 30 min STs with a 4-h interval. Postural sway was measured for 30 s at 0 h, 30 min, 1 h, 2 h, 3 h, and 4 h during the PST. In Session 2, optimal intensity nGVS was applied to subjects for 3 h and the subjects were monitored after the cessation of the stimulus for 4 h during the PST. Postural sway was measured for 30 s at 1 and 2 h during the ST and at 0 h, 30 min, 1 h, 2 h, 3 h, and 4 h during the PST. Subjects were allowed to act freely in the hospital during and after the stimulation. The subjects were randomly allocated by a permuted-block design at a 1:1 ratio, block size 2 (Session 1 followed by Session 2, or Session 2 followed by Session 1). The random allocation sequence was generated by Clinical Research Support Center at the University of Tokyo Hospital. All analyses for this article were performed by the independent academic biostaticians. Enrollment was conducted by the investigators.

### Data analysis

Data were shown as mean ± standard deviation. Statistical analysis was performed using the SAS software (Version 9.4; SAS Inc., Cary, NC, USA). We assessed the normality of data by visual inspection and the Kolmogorov-Smirnov test, and the homoscedasticity by Bartlett test. If P < 0.1 for these tests, we considered that the data showed non-normality or heteroscedasticity. First, we analyzed the association between original postural instability and the presence of an optimal intensity of nGVS. The optimal intensity of nGVS was determined before each session and the subjects were divided into three groups on the basis of whether or not an optimal intensity could be determined: (1) subjects in which an optimal intensity could be determined before both sessions (“Twice”)(n = 40: 20 subjects, 2 sessions each), (2) subjects who had an optimal intensity before only one of the two sessions (“Once”)(n = 9, 7 subjects with one session each, 2 subjects with one session only due to device failure), and (3) subjects for whom an optimal intensity could not be determined before either session (“Neither”)(n = 2, one subject, 2 sessions). Supplementary Table S1 shows the baseline value (i.e. the value in the eyes-closed/foam rubber condition without nGVS) and the ratio of the baseline value to the value in the eyes-open condition without nGVS (“eyes-closed foam ratio”), for each group. For the ‘once’ group, original postural instability is described separately for the measurement periods with and without an optimal intensity. We also divided the individual measurement periods of all subjects into two groups: measurement periods with an optimal intensity (n = 49) and measurement periods without an optimal intensity (n = 9), and compared original postural instability between the two groups using box-and-whisker plot and Mann-Whitney U test or t-test depending on its distribution. Next, we analyzed the association between original postural instability and the effect of nGVS on postural stability during the 3-h stimulation, for those who had an optimal intensity before both sessions. We defined the IR (%) as 100 (1 – normalised ratio), where the normalised ratio is the ratio of the value at the measurement period to that at baseline. We calculated Pearson’s correlation coefficient to explore the association between IR at ST 1 h and 2 h and the baseline value and the association between IR and the eyes-closed foam ratio. Then, we conducted mixed-effects model analysis using IR at ST 1 h and 2 h as outcome, time (categorical) and the baseline value as fixed-effects, and intercept by subjects as random-effects. The model utilizes “random effect” term, which accounts for repeated measures within same subjects by assuming a compound symmetry structure for error term. The factor time has 2 levels, 1 h and 2 h. We also used the eyes-closed foam ratio as a fixed effect instead of the baseline value. To address whether anthropometrical factors affect posturographic data, we added heights and weights in the fixed factors. If P < 0.1 for these factors, we included these factors in the mixed-effects model analysis. Finally, we analyzed the association between original postural instability and the long-term post-stimulation effect of nGVS on postural stability, for those who had an optimal intensity before both sessions. Although the effect of nGVS might be affected by the duration of the nGVS, the correlation is not strong in our previous paper^1^. Therefore, we did not focus on their relationship here. Rather, we analysed both posturographic data at PST 30 min, 1 h, 2 h and 3 h in the first 30-min nGVS and in the 3-h nGVS simultaneously. Data in the second 30-min nGVS were not used due to the influence of the cumulative effect. We calculated Pearson’s correlation coefficient to explore the association between the IR at PST 30 min, 1 h, 2 h and 3 h and the baseline value and the association between IR and the eyes-closed foam ratio. We conducted mixed-effects model analysis using IR at PST 30 min, 1 h, 2 h and 3 h in the first 30-min nGVS and in the 3-h nGVS as outcome, time, session, and interaction between time and session (categorical) and the baseline value as fixed-effects, and intercept by subjects as random-effects. The factor session has 2 levels, 1 and 2, and the factor time has 4 levels, 30 min, 1 h, 2 h and 3 h. We also used the eyes-closed foam ratio as a fixed effect instead of the baseline value. To address whether anthropometrical factors affect posturographic data, we added heights and weights in the fixed factors. If P < 0.1 for these factors, we included these factors in the mixed-effects model analysis. P < 0.05 was regarded as statistically significant. Due to the exploratory nature of the present study, we did not adjust for multiple testing.

Statistical Analysis conducted by Takuya Kawahara MPH and Yukari Uemura PhD (Biostatistics Division, Clinical Research Support Center, The University of Tokyo Hospital)

## Supplementary information

Supplementary Table S1
