# Supplementary material for: Noisy galvanic vestibular stimulation has a greater ameliorating effect on posture in unstable subjects: a feasibility study
Source: Sci Rep. 2019 Nov 20;9:17189. doi: 10.1038/s41598-019-53834-7 (PMC6868214; doi:10.1038/s41598-019-53834-7)
Supplement: Supplementary file 1 — Supplementary Table S1 [file 41598_2019_53834_MOESM1_ESM.pdf]

Noisy galvanic vestibular stimulation has a greater ameliorating effect on posture in unstable subjects: a feasibility study.

Authors: [*1, 2]Chisato Fujimoto MD PhD, [1]Makoto Kinoshita MD PhD, [1]Teru Kamogashira MD PhD, [1]Naoya Egami MD PhD, [3]Takuya Kawahara MPH, [3]Yukari Uemura PhD, [4]Yoshiharu Yamamoto PhD, [1]Tatsuya Yamasoba MD PhD and [1]Shinichi Iwasaki MD PhD

[1]Department of Otolaryngology and Head and Neck Surgery, Graduate School of Medicine, The University of Tokyo 7-3-1, Hongo, Bunkyo-ku, Tokyo 113-8655, Japan (C.F., M.K., T.K., N.E., T.Y., S.I.)

[2]Department of Otolaryngology, Tokyo Teishin Hospital, 2-14-23, Fujimi, Chiyoda-ku, Tokyo, 102-8798, Japan (C.F.)

[3]Biostatistics Division, Clinical Research Support Center, The University of Tokyo Hospital 7-3-1, Hongo, Bunkyo-ku, Tokyo, 113-8655, Japan (T.K., Y.U.)

[4]Educational Physiology Laboratory, Graduate School of Education, The University of Tokyo 7-3-1, Hongo, Bunkyo-ku, Tokyo 113-0033, Japan (Y.Y.)

Corresponding Author: Chisato Fujimoto MD PhD

Department of Otolaryngology and Head and Neck Surgery, Graduate School of Medicine, The University of Tokyo 7-3-1, Hongo, Bunkyo-ku, Tokyo 113-8655, Japan

Telephone number: +81-3-5800-8665, Fax number: +81-3-3814-9486,

E-mail: cfujimoto-tky@umin.ac.jp

Co-authors: Makoto Kinoshita MD PhD (kinoshitam-zao@umin.ac.jp), Teru

Kamogashira MD PhD (tkamogashira-tky@umin.ac.jp), Naoya Egami MD PhD (n-egami@umin.ac.jp), Takuya Kawahara MPH (tkawahara-tky@umin.ac.jp), Yukari Uemura PhD (yukariuemura-tky@umin.ac.jp), Yoshiharu Yamamoto PhD (yamamoto@p.u-tokyo.ac.jp), Tatsuya Yamasoba MD PhD (tyamasoba-tky@umin.ac.jp), Shinichi Iwasaki MD PhD (iwashin-tky@umin.ac.jp)

Statistical Analysis conducted by Takuya Kawahara MPH and Yukari Uemura PhD (Biostatistics Division, Clinical Research Support Center, The University of Tokyo Hospital)

Supplementary Table S1 Descriptive statistics of the original postural instability stratified by the patterns of the presence of the optimal intensity.

	Parameter	Presence of the optimal intensity		n*	Mean	SD	Min	Median	Max
Velocity	Baseline value (cm / 30 s)	Twice (*N* = 20)#		40	219.9	61.1	136.4	210.5	406.5
		Once (N = 9)†	with optimal intensity	9	181.5	41.5	132.1	189.5	234.7
			without optimal intensity	7	145.6	29.3	110.2	141.7	197.0
		Neither (*N* = 1)		2	246.8	23.2	230.5	246.8	263.2
	Eyes-closed foam ratio	Twice (*N* = 20)		40	2.22	0.78	1.03	2.18	5.10
		Once (N = 9)†	with optimal intensity	9	2.22	0.41	1.54	2.14	2.87
			without optimal intensity	7	1.70	0.29	1.40	1.69	2.28
		Neither (*N* = 1)		2	1.81	0.77	1.26	1.81	2.35
Area	Baseline value (cm^2)	Twice (*N* = 20)#		40	18.4	6.6	8.3	16.6	36.0
		Once (N = 9)†	with optimal intensity	9	14.2	5.2	7.2	13.6	22.1
			without optimal intensity	7	8.7	3.6	4.0	9.5	13.4
		Neither (*N* = 1)		2	17.6	2.8	15.7	17.6	19.6
	Eyes-closed foam ratio	Twice (*N* = 20)		40	3.99	2.48	1.24	3.60	15.48
		Once (N = 9)†	with optimal intensity	9	3.84	1.03	2.33	3.88	5.25
			without optimal intensity	7	2.46	0.96	1.19	2.47	4.11
		Neither (*N* = 1)		2	3.01	1.81	1.73	3.01	4.29
RMS	Baseline value (cm)	Twice (*N* = 20)#		40	1.8	0.3	1.2	1.8	2.4
		Once (N = 9)†	with optimal intensity	9	1.5	0.3	1.1	1.6	1.9
			without optimal intensity	7	1.2	0.3	0.9	1.3	1.5
		Neither (*N* = 1)		2	1.7	0.1	1.6	1.7	1.8
	Eyes-closed foam ratio	Twice (*N* = 20)		40	1.89	0.52	0.98	1.85	3.96
		Once (N = 9)†	with optimal intensity	9	1.89	0.25	1.46	1.90	2.39
			without optimal intensity	7	1.52	0.29	1.07	1.58	1.91
		Neither (*N* = 1)		2	1.66	0.50	1.31	1.66	2.01

* Number of measurements. # Twenty had the optimal intensity at both measurement times. † Nine had the optimal intensity at one measurement time. Out of them, two could not have the data of optimal intensity due to failure of the device at the other measurement time (before Session 2). RMS, root mean square, SD, standard deviation.
